# Supplementary material for: Effect of Replacement of Synthetic vs. Natural Curing Agents on Quality Characteristics of Cinta Senese Frankfurter-Type Sausage
Source: Animals (Basel). 2019 Dec 19;10(1):14. doi: 10.3390/ani10010014 (PMC7022300; doi:10.3390/ani10010014)
Supplement: Supplementary file 1 [file animals-10-00014-s001.pdf]

animals

MDPI

Supplementary table 1. List of attributes of sausage sensory profile.

Attributes	Definition
Colour uniformity	Presence of not homogeneity in the pieces
Lightness	Intensity of the characteristic colour of the sausage (dark–light)
Odour	Intensity of overall odour before eating the sample
Off odour	Level of odour not associated with those characteristic
Flavour	Intensity of overall flavour
Off flavour	Level of flavour not associated with those characteristic
Bitter taste	Intensity of bitterness during eating the sample
Tannic taste	Astringency degree during eating the sample
Juiciness	Moisture of the product in the mouth during early chewing
Hardness	Force required to bite the sausage and to make the sample ready to be swallowed
Overall acceptability	Overall liking in terms of sensory inputs (appearance, taste/flavour and texture)
